# Association of frequent intake of trans fatty acids and saturated fatty acids in diets with increased susceptibility of atopic dermatitis exacerbation in young Chinese adults: A cross‐sectional study in Singapore/Malaysia

**DOI:** 10.1002/ski2.330

**Published:** 2024-06-26

**Authors:** Jun Jie Lim, Sing Wei Lim, Kavita Reginald, Yee‐How Say, Mei Hui Liu, Fook Tim Chew

**Affiliations:** ^1^ Department of Biological Sciences Faculty of Science National University of Singapore Singapore Singapore; ^2^ Department of Biological Sciences School of Medicine and Life Sciences Sunway University Petaling Jaya Malaysia; ^3^ Department of Biomedical Science Faculty of Science Universiti Tunku Abdul Rahman (UTAR) Kampar Malaysia; ^4^ Department of Food Science & Technology Faculty of Science National University of Singapore Singapore Singapore

## Abstract

**Background & Objective:**

Numerous evidence has attributed diets with a high fatty acids (FAs) intake to be associated with atopic dermatitis (AD) development. Therefore, this study investigated the association between intake frequencies of five dietary FAs and AD exacerbations among young Chinese adults from Singapore and Malaysia.

**Methods:**

A validated International Study of Asthma and Allergies in Childhood (ISAAC) questionnaire was investigator‐administered to 13,561 subjects to collect information on socioeconomic, anthropometric, dietary and lifestyles habits, and personal/family medical histories. Six novel dietary indices were derived to analyse the associations between total FAs, trans fatty acids (TFAs), saturated fatty acids (SFAs), monounsaturated fatty acids, linoleic acids, and alpha‐linolenic acids in diets and AD exacerbation. Synergy factor (SF) analysis was used to identify interactions between the dietary FAs to influence disease susceptibility.

**Results:**

In our multivariable model adjusted for age, gender, BMI, parental eczema, and lifestyle factors, a diet high in total estimated FAs was strongly associated with AD (Adjusted Odds Ratio (AOR): 1.227; 95% Confidence Interval (CI): 1.054–1.429; adjusted *p*‐value <0.01). Particularly, high estimated total TFAs and SFAs were significantly associated with AD exacerbations including chronic and current moderate‐to‐severe AD. The association between TFAs and AD remained strong even controlled for the total FAs in diets and false discovery rate corrected (AOR: 1.516; 95% CI: 1.094–2.097; adjusted *p*‐value <0.05). Similarly, having a high SFAs in diets was associated with AD (AOR: 1.581; 95% CI: 1.106–2.256; adjusted *p*‐value <0.05) independently on the total FAs in diets. FAs in diets do not interact to influence AD.

**Conclusion:**

Overall, these results highlighted an association between high dietary TFAs and SFAs and AD exacerbations in an Asian population.

## Graphical abstract

Focussing on a large epidemiologically and clinically well‐defined allergic cohort of 13,561 young Chinese adults in Singapore and Malaysia, we highlighted a significant association between fatty acids (FAs) in diets and atopic dermatitis (AD). Excessive dietary intake of saturated and trans fatty acids was strongly associated with a higher risk for chronic and moderate‐to‐severe AD. Notably, this risk association was independent on the total FAs intake in diets and other potential confounders such as lifestyle habits and genetics.

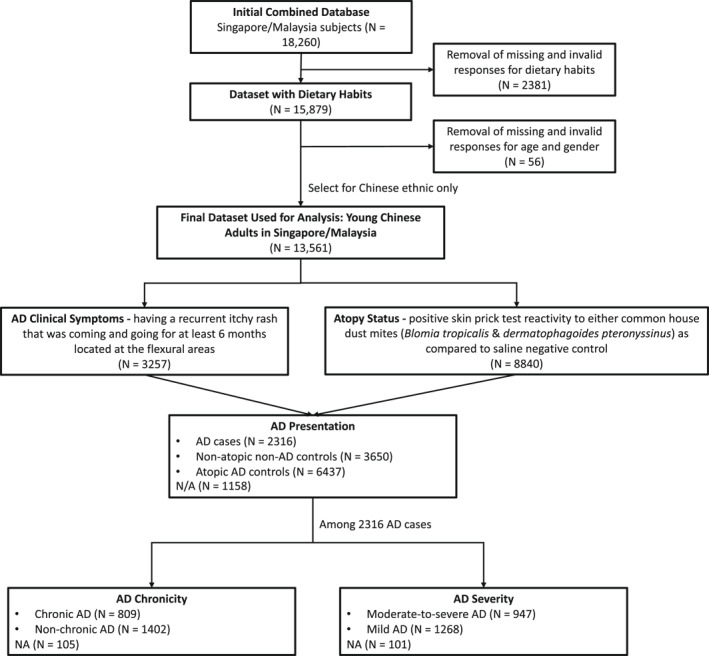

**What is already known about this topic?**

- Dietary habits have been increasingly associated with atopic dermatitis (AD). Specifically, excessive intake of fatty acids (FAs) induces inflammation and worsened AD conditions.
- However, regional epidemiology studies to characterise the specific types of dietary FAs and AD exacerbations are lacking.

**What does this study add?**

- The study adds evidence to the associations between frequent intake of various FAs in diets and AD exacerbations.
- The first regional study in Asia to provide insights into the potential role of dietary factors in the development and exacerbation of AD.

## 1 INTRODUCTION

Atopic dermatitis (AD) is a chronic, itchy inflammatory skin disease that typically manifests in early childhood, but AD can persist into adulthood and largely affects the patient's quality of life and productivity.^1^ Although AD has a strong genetic component, there is emerging epidemiological evidence that suggests the importance of diet and lifestyle choices in influencing AD development.^2^, ^3^ Recently, considerable literature has grown up around the theme of dietary patterns and nutrition with AD prevalence in developed nations.^4^, ^5^ The International Study of Asthma and Allergies in Childhood (ISAAC) phase III study highlighted that a frequent intake of fast food, butter, and margarine is positively associated with increased symptom prevalence of AD in adolescents.^6^, ^7^

Of these, fatty acids (FAs) are of key interest because of their immunoregulatory effects. Frequent intake of high levels of FAs promotes low‐grade chronic systemic inflammation and may catalyze AD development.^8^ This association between FAs and AD, however, is complex and controversial. While some studies suggest an association between FAs supplementation and improved AD symptoms among predisposed individuals with deficiencies in FAs,^9^, ^10^ there are also evidence to highlight the potential pathological effects of FAs associated with AD.^11^, ^12^ Therefore, continued research and a more in‐depth investigation into specific dietary FAs are crucial for a comprehensive understanding of this intricate relationship. Moreover, most large‐scale studies on AD with FAs are largely limited to Western paediatric populations with very little focus on the Asia adult population. Given that dietary habits in Asia are very distinct from the West, there is a need for detailed diet association studies that would be relevant to an Asian context. Within the vast and culturally diverse continent of Asia, dietary habits vary widely and they are shaped by a multitude of factors.^13^ For the interest of this study, we focused on the Chinese to provide a more targeted and in‐depth analysis that is representative of a significant segment of the Singapore and Malaysia population.

We previously reported in our cross‐sectional study that a high total estimated fat amount in diets was strongly associated with various atopic diseases, which include AD.^14^ There was still uncertainty, however, regarding whether different fat types have distinctive influences on AD development. Even among foods with similar total fat composition, the FAs profiles can differ significantly. Thus, the specific objective of this study was to investigate the association between intake frequencies of various FAs in diets and AD exacerbation (chronic and moderate‐to‐severe AD) using our expanded Singapore/Malaysia Cross‐sectional Genetics Epidemiology Study (SMCGES) cohort. Here, we focused on examining five dietary FAs commonly derived from foods which comprise trans fatty acids (TFAs), SFAs, monounsaturated fatty acids (MUFAs), linoleic acids (LAs) and alpha‐linolenic acids (ALAs).^15^ We expect that a better understanding of the relationship between AD and various FAs intakes can identify potential modifiable dietary factors that exacerbate or improve AD symptoms and is important for disease management. Furthermore, understanding the role of dietary FAs in AD can inform dietary recommendations for individuals with AD. Ultimately, the insights from this study provide a basis for the development of targeted dietary interventions that can improve AD exacerbation.

## 2 METHODS AND MATERIALS

### 2.1 Study population and disease definition

During the initial stage of the cross‐sectional study, we aimed to obtain a representative sampling of the general population. This baseline information captured is essential for understanding the natural distribution of AD in the Singapore and Malaysia landscape with minimised selection bias. Subjects were sampled randomly, unbiasedly, and consecutively from an epidemiology collection between 2005 and 2022, composed mainly of university students (mean age 22.51, standard deviation ± 5.90) from the National University of Singapore, Sunway University and Universiti Tunku Abdul Rahman. Upon enrolment in the study, subjects were asked to voluntarily indicate their ethnic identity and verification was conducted by cross‐referencing with subjects' official identification documents. Furthermore, as this study is also a parallel genetic study, all subjects were fully genome‐wide genotyped. The whole genome genotype were cross referenced to reference population and they did not differ significantly.^16^ As this study follows a cross‐sectional design, each subject was restricted to enrolling in the study only once at any given time. Moreover, subjects who had been using anti‐histamines or other related drugs within the 3 days preceding data collection were strongly encouraged to discontinue their medication before participating in the study. This precautionary measure was taken to ensure the accuracy of allergy assessment with the skin prick test (SPT). A standardized ISAAC questionnaire was investigator‐administered to the subjects to collect information on their sociodemographic, dietary and lifestyles habits, and personal/family medical histories.

**FIGURE 1:**
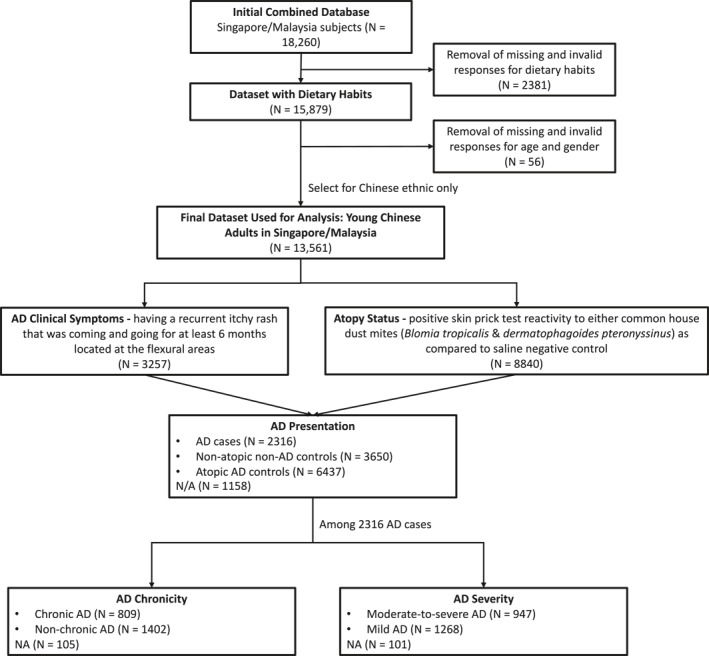
Flowchart summarising subjects' atopic dermatitis disease status.

As compared to our earlier studies conducted before 2019,^3^, ^14^ the total population size was expanded from 16,336 to 18,260 in our ongoing epidemiology collections. 2437 subjects were subsequently excluded due to missing or invalid data for age, gender, race, and dietary habits. As a large proportion of Singapore's population constitutes ethnic Chinese,^17^ 13,561 Chinese were selected to form our final dataset (Figure 1). This ensures that the dataset is representative of the SMCGES population and minimises ascertainment bias that affects the reliability of subsequent analyses. A separate power calculation as described by Charan and Biswas^18^ was performed to estimate the sample size appropriate for our cross‐sectional study. We have assumed a significance level of 0.05 and an estimated proportion between 5% and 20%. Remarkably, our study have included a substantially large sample of 13,561 subjects that fulfiled the minimal sample size in ensuring a high level of statistical level of power. This enhances the robustness and precision of our findings and enable us to provide a representative analysis of the target population.

In this study, AD subjects were determined have a positive SPT response towards either of the two common mite allergens (*Blomia tropicalis* and *Dermatophagoides pteronyssinus*) and have a recurrent itchy rash for at least 6 months specifically in the flexural regions including areas under the buttocks, around the neck, cheeks, ears, or eyes. AD symptoms defined in this study followed the validated guidelines of the UK Working Party's diagnostic criteria and Hanifin and Rajka criteria.^19^, ^20^ Concurrently, the subjects were assessed by our trained personnel for the presence of an itchy flexural rash. These assessments were cross validated periodically by a dermatologist and found to be concordant. Non‐atopic non‐AD controls were determined to be having a negative SPT response and not having the recurrent itchy flexural rash. To assess AD exacerbations, we considered both severity and chronicity to offer a more comprehensive approach to understanding the specific characteristics that are implicated in subjects with exacerbated AD conditions. AD severity accounts for the intensity of AD symptoms in terms of the average frequency of sleep disturbance at night caused by the itchy rash in the last 12 months. AD chronicity accounts for the duration of the AD symptoms in terms of the completeness of itchy rash clearance in the last 12 months. The assessment of AD severity and chronicity has been extensively validated by ISAAC.^21^ In summary, we have identified 3650 non‐atopic non‐AD controls, 2316 AD cases with 947 of them having moderate‐to‐severe AD and 809 having chronic AD (Figure 1). Please refer to Lim *et al*. for the detailed SPT and AD diagnosis criteria.^3^, ^14^

### 2.2 Estimation of dietary fatty acids intake

The questionnaire included a modified section from ISAAC Phase III study to examine dietary habits based on the intake frequency of 16 food types, on average, in the past 12 months^6^ The 16 food types are meat (including beef, chicken, pork, lamb), seafood (including fish), margarine, butter, eggs, pasta, fruits, vegetables (green and root), pulses (peas, beans, lentils), nuts, milk, Yakult®/Vitagen®/other similar yoghurt drinks, burgers/fast food, cereals (including bread), rice, and potatoes. The intake frequencies were listed to be i) never or occasionally, ii) once or twice per week, and iii) most or all days.

Following, we derived six novel dietary indices to assess the estimated total FAs in the weekly diets of 13,561 Chinese subjects. These dietary indices account for the intake frequencies and average portion size (100 g/serving). With reference to the rubrics reported by Manousos *et al*., a corresponding score was assigned to each intake frequency response—0 (never or occasionally), 2 (once or twice per week), 7 (most or all days).^22^ For the interests of this study, we employed a methodology that involves averaging the estimated amounts of FAs derived from a list of common food items within each food type. The nutritional information on the estimated FAs amounts (100 g per serving of a given food type) was obtained from the United States Department of Agriculture (USDA) FoodData Central database (Table S1). This approach aims to provide a representative estimation of FAs intake, accounting for the diversity within specific food categories. Adequate sensitivity analyses were conducted to ensure robustness in the design of the dietary indices. From the preliminary distributional assessment of our SMCGES cohort (*N* = 13, 561), a cut‐off based on the 33rd and 66th percentiles was used to categorise the subjects into the low total estimated amount and high total estimated amount groups, respectively (Table S2).

### 2.3 Statistical analysis

Data entry and processing was done using Microsoft Excel (http://office.microsoft.com/en‐us/excel/), and further analysis was performed using the R statistical programming language (RStudio Team, 2021). Logistic regression was used to calculate odds ratio (OR) for the associations between disease outcomes and intake frequencies of total estimated FAs. Potential confounding variables were adjusted in subsequent multivariable analyses with values represented as adjusted odds ratios (AORs), 95% confidence intervals (CI), and *p*‐values. To ensure the reliability of our statistical findings, false discovery rate (FDR) correction was applied to control for multiple comparisons.^23^ A synergy factor (SF) analysis, as described by Cortina‐Borja *et al*.,^24^ was performed to identify the presence of any synergistic/antagonistic interactions between the dietary FAs in their ability to influence AD disease outcome. All analyses with *p*‐value <0.05 were statistically significant.

## 3 RESULTS

### 3.1 Population characteristics

AD prevalence was high (17.1%) among 13 561 Chinese subjects, with fewer AD cases having chronic (5.97%) and moderate‐to‐severe AD (6.98%) as compared to non‐chronic (10.3%) and mild AD (9.35%), respectively (Figure 1). In contrast with the previous findings,^3^ there was a rise in the AD prevalence and this was not unexpected as AD prevalence was projected to increase in urbanised Asian countries.^25^ Overall, there were more female than male subjects across the disease groups, with the largest male: female ratio (1:1.5) among moderate‐to‐severe AD (Table 1). Of the disease groups, the proportion of having parental history of AD was the highest among those with moderate‐to‐severe AD. Although most subjects spent >1 h/day on TV/computer, almost two‐thirds of them engaged in active physical activities frequently. More than half the subjects across the disease groups have a healthy body mass index (BMI) range (18.0–23.0 kg/m^2^) and consumed alcohol occasionally. The prevalence of lower education is more common across the disease groups. As lifestyles, genetic and socioeconomic factors have been suggested to be strong risk factors for AD, confounding variables such as age, gender, BMI, parental AD, use of TV/computer, engagement in physical activities, use of alcohol, and parental education were altogether controlled in the subsequent multivariable logistic regression analyses.^2^, ^3^

**TABLE 1:** Demographics of the 13,561 young Chinese adults from the Singapore/Malaysia Cross‐sectional Genetics Epidemiology Study (SMCGES) cohort.

Variables	Atopic dermatitis (AD)^a^		
	AD (*N* = 2316)	Moderate‐to‐severe AD (*N* = 947)	Chronic AD (*N* = 809)
Gender			
Male	963 (41.6%)	370 (39.1%)	331 (40.9%)
Female	1353 (58.4%)	577 (60.9%)	478 (59.1%)
Parental AD^b^			
No	1774 (76.6%)	706 (74.6%)	601 (74.3%)
Yes	523 (22.6%)	237 (25.0%)	204 (25.2%)
N/A	19	4	4
Body Mass index, asian class (kg/m^2^)			
Healthy (18.5‐23.0)	1207 (52.1%)	479 (50.6%)	430 (53.2%)
Underweight (<18.5)	391 (16.9%)	164 (17.3%)	122 (15.1%)
Overweight (>23.0)	411 (17.7%)	181 (19.1%)	149 (18.4%)
N/A	307	123	108
Active physical activities (times/week)			
Never or only occasionally	765 (33.0%)	329 (34.7%)	276 (34.1%)
Once or twice per week	1223 (52.8%)	488 (51.5%)	420 (51.9%)
Most or all days	311 (13.4%)	126 (13.3%)	104 (12.9%)
N/A	17	4	9
Sedentary lifestyles ‐ TV/computer usage (hours/day)			
Less than 1 h	313 (13.5%)	115 (12.1%)	94 (11.6%)
1–3 h	720 (31.1%)	279 (29.5%)	274 (33.9%)
More than 3 h–5 h	606 (26.2%)	257 (27.1%)	194 (24.0%)
More than 5 h	669 (28.9%)	293 (30.9%)	243 (30.0%)
N/A	8	3	4
Use of alcohol			
Non‐drinker	941 (40.6%)	395 (41.7%)	346 (42.8%)
Occasional	1190 (51.4%)	479 (50.6%)	403 (49.8%)
Frequent	48 (2.10%)	21 (2.20%)	19 (2.30%)
N/A	137	52	41
Parental education^c^			
Lower	1027 (44.3%)	382 (47.2%)	424 (44.8%)
Middle	580 (25.0%)	198 (24.5%)	241 (25.4%)
Higher	628 (27.1%)	198 (24.5%)	253 (26.7%)
N/A	81	31	29

^a^ Atopic AD is defined as a subject with positive skin prick test (SPT) reaction to either one of the two common house dust mites (*Blomia tropicalis* and *Dermatophagoides pteronyssinus*) and having the AD clinical symptoms of an itchy rash that is coming and going for at least 6 months in the flexural areas.

^b^ Parental eczema is defined by the presence of either paternal and/or maternal eczema from the immediate family.

^c^ A lower parental education refers to having an educational background equivalent to secondary education or lower. A middle parental education refers to individuals with at least one 1 parent with an educational background equivalent to a tertiary education. Both parents having a tertiary educational background is considered to be having a higher education.

### 3.2 Intake of various estimated fatty acids in diets

Currently, there are insufficient data to establish a specific recommended dietary allowance (RDA) for FAs in adults. Based on the general recommendation by World Health Organisation for normal adults (55–65 g/day for a typical 2000 kcal diet), the estimated consumption of total FAs was high among the SMCGES subjects (mean 539.1 g/week) (Table S2).^26^ The Singapore's 2018 National Nutrition Survey highlighted that Singaporeans were consuming more fats in their diets when compared to 2010. However, there was a gradual improvement in dietary fat quality with SFAs consumption reduced to 36.0% of the total fat proportion in diets.^27^, ^28^ Similarly, the estimated total SFAs consumption formed 35.5% of the total fat proportion in diets among the SCMGES subjects. The mean estimated total LAs and MUFAs intake in diets were at 75.72 g/week and 216.9 g/week, respectively. While the mean estimated total ALAs (7.993 g/week) and TFAs (18.976 g/week) was comparatively lower, TFAs intake was alarmingly higher than the ideal recommendation (<1.0% of total daily energy intake).^29^

### 3.3 Diets with high total estimated trans fatty acids and saturated fatty acids intake accentuated associated risks for AD exacerbation

The multivariable logistic model revealed a positive association between a high total estimated FAs in diets and all AD phenotypes, including exacerbated AD (Table 2). Furthermore, a dose‐dependent response was observed in the AORs with increased total estimated FAs in diets.

**TABLE 2:** Associations between intake frequencies of various dietary fatty acids and atopic dermatitis (AD), chronic AD, and current moderate‐to‐severe AD among 13,561 young Chinese adults from the Singapore/Malaysia Cross‐sectional Genetics Epidemiology Study (SMCGES) cohort.

	AD presentation			AD exacerbations					
				Chronic AD^a^			Moderate‐to‐severe AD^b^		
	AOR^c^	95% CI	Adjusted p^c^	AOR^c^	95% CI	Adjusted p^c^	AOR^c^	95% CI	Adjusted p^c^
i) Total fatty acids									
Low estimated total fatty acids amount (*N* = 4475)	1.000	REF	‐	1.000	REF	‐	1.000	REF	‐
Moderate estimated total fatty acids amount (*N* = 4475)	1.093	0.940–1.271	0.246	1.053	0.844–1.315	0.647	1.103	0.897–1.356	0.352
High estimated total fatty acids amount (*N* = 4611)	1.227	1.054–1.429	0.008*	1.168	0.935–1.461	0.171	1.227	0.997–1.510	0.053
ii) Tran fatty acids (TFAs)									
Low estimated total TFA amount (*N* = 4490)	1.000	REF	‐	1.000	REF	‐	1.000	REF	‐
Moderate estimated total TFA amount (*N* = 4479)	1.129	0.971–1.312	0.114	1.074	0.860–1.342	0.529	1.207	0.983–1.483	0.073
High estimated total TFA amount (*N* = 4592)	1.268	1.088–1.478	0.002*	1.262	1.009–1.578	**0.041**	1.287	1.044–1.587	**0.018**
iii) Saturated fatty acids (SFAs)									
Low estimated total SFA amount (*N* = 4476)	1.000	REF	‐	1.000	REF	‐	1.000	REF	‐
Moderate estimated total SFA amount (*N* = 4456)	1.061	0.912–1.234	0.445	0.995	0.794–1.246	0.965	1.018	0.827–1.253	0.868
High estimated total SFA amount (*N* = 4629)	1.276	1.096–1.486	0.002*	1.291	1.035–1.612	**0.024**	1.253	1.020–1.540	**0.032**
iv) Monounsaturated fatty acids (MUFAs)									
Low estimated total MFUA amount (*N* = 4475)	1.000	REF	‐	1.000	REF	‐	1.000	REF	‐
Moderate estimated total MFUA amount (*N* = 4475)	0.997	0.857–1.159	0.965	1.024	0.820–1.279	0.837	0.985	0.801–1.211	0.884
High estimated total MFUA amount (*N* = 4611)	1.164	1.000–1.356	**0.049**	1.150	0.919–1.438	0.222	1.161	0.945–1.426	0.156
v) Linoleic acids (LAs)									
Low estimated total LA amount (*N* = 4475)	1.000	REF	‐	1.000	REF	‐	1.000	REF	‐
Moderate estimated total LA amount (*N* = 4475)	0.961	0.827–1.118	0.610	0.936	0.749–1.169	0.560	0.999	0.813–1.229	0.996
High estimated total LA amount (*N* = 4611)	1.095	0.941–1.273	0.241	1.062	0.851–1.326	0.592	1.138	0.927–1.399	0.218
vi) Alpha‐linolenic acids (ALAs)									
Low estimated total ALA amount (*N* = 4479)	1.000	REF	‐	1.000	REF	‐	1.000	REF	‐
Moderate estimated total ALA amount (*N* = 4471)	1.110	0.955–1.290	0.175	1.032	0.826–1.289	0.784	1.141	0.929–1.401	0.208
High estimated total ALA amount (*N* = 4611)	1.163	0.998–1.354	0.053	1.176	0.942–1.469	0.153	1.171	0.951–1.442	0.138

*Note*: All disease cases were compared to the non‐atopic non‐AD controls which were subjects with negative skin prick test response to two common house dust mites and absence of a recurrent itchy rash in flexural areas.

Abbreviations: AD, atopic dermatitis; ALA, alpha‐linolenic acids; AOR, adjusted odds ratios; CI, confidence intervals; LA, linoleic acid; MUFA, monounsaturated fatty acids; SFA, saturated fatty acids; TFA, trans fatty acids.

^a^ Chronic AD was defined as a subject with a recurrent itchy rash in flexural areas that was not cleared completely during the past 12 months.

^b^ Moderate‐to‐severe AD was defined as a subject with a recurrent itchy rash in flexural areas that experienced sleep disturbances at night in the past 12 months.

^c^ Multivariable logistic regression analysis was controlled for potential confounding effects from age, gender, BMI, parental eczema, use of alcohol, engagement in physical activities, use of TV/computer, and parental education.

^4^ *P*‐value obtained from logistic regression has been adjusted by False discovery rate (FDR) correction. *P* < 0.05 was considered statistically significant. Unadjusted *p* < 0.05 was bolded while adjusted *p* < 0.05 was further indicated with an asterisk.

Most importantly, the multivariable model with the FDR adjusted *p*‐value highlighted that a high estimated total TFAs were strongly associated with AD (AOR: 1.268) and moderate‐to‐severe AD (AOR: 1.287), respectively (Table 2). This indicated that frequent intake of TFAs in diets imposed stronger associated risks for AD exacerbation than the other FAs. Similarly, a high estimated total SFAs imposed strongly associated risks for AD even after FDR correction (AOR: 1.276). In the multivariable model corrected by FDR, increasing MUFAs, LAs, and ALAs in diets were not associated with AD and AD exacerbations.

These findings, however, must be approached with some caution as diets with a high total estimated FAs may confound the analysis of the specific effect of other individual FAs. Thus, we stratified subjects further based on their total FAs intake to ensure the specific association for each FAs was reliable and not overestimated (Table 3). The intake of a high total estimated TFAs was significantly associated with AD (AOR: 1.516) independently on total FAs. While a high total estimated SFAs was strongly associated with both AD (AOR: 1.581) and chronic AD (AOR: 1.929) independently on total FAs. While only a diet high in both total FAs and MUFAs was associated with AD (AOR: 1.214). This indicated that the association between intake of MUFAs in diets and AD was dependent on the total FAs. Interestingly, diets with high LAs were associated with AD (AOR: 1.579) and was independent on total FAs. Overall, the differences in associated AORs suggested that the individual dietary FAs have different roles and are implicated in varying degrees in promoting AD exacerbations, with stronger associations observed between AD exacerbations, TFAs and SFAs.

**TABLE 3:** Association between intake of i) trans fatty acids, ii) saturated fatty acids, iii) monounsaturated fatty acids, iv) linoleic acid, and v) alpha‐linoleic acid and various atopic dermatitis (AD) phenotypes among 13,561 young Chinese subjects with varying intake of total fatty acids in diets.

	AD			Chronic AD			Moderate‐to‐severe AD		
	AOR^a^	95% CI	Adjusted p^c^	AOR^a^	95% CI	Adjusted p^c^	AOR^a^	95% CI	Adjusted p^c^
i) Trans fatty acids (TFAs)									
Low estimated total TFA & low estimated total fat (*N* = 3861)	1.000	REF	‐	1.000	REF	‐	1.000	REF	‐
Low estimated total TFA^b^ & high estimated total fat (*N* = 630)	0.863	0.632–1.168	0.345	0.809	0.498–1.269	0.371	0.735	0.460–1.137	0.181
High estimated total TFA^b^ & low estimated total fat (*N* = 579)	1.516	1.094–2.097	0.012*	1.524	0.941–2.405	0.077	1.516	0.974–2.317	0.059
High estimated total TFA & high estimated total fat (*N* = 4013)	1.206	1.023–1.421	**0.026**	1.187	0.934–1.509	0.162	1.197	0.956–1.499	0.117
ii) Saturated fatty acids (SFAs)									
Low estimated total SFA & low estimated total fat (*N* = 4206)	1.000	REF	‐	1.000	REF	‐	1.000	REF	‐
Low estimated total SFA^b^ & high estimated total fat (*N* = 270)	1.147	0.731–1.775	0.543	1.118	0.562–2.074	0.736	1.336	0.738–2.312	0.318
High estimated total SFA^b^ & low estimated total fat (*N* = 434)	1.581	1.106–2.256	0.012*	1.929	1.181–3.075	**0.007**	1.460	0.886–2.338	0.124
High estimated total SFA & high estimated total fat (*N* = 4195)	1.259	1.074–1.476	0.005*	1.241	0.984–1.565	0.069	1.260	1.016–1.563	**0.035**
iii) Monounsaturated fatty acids (MUFAs)									
Low estimated total MUFA & low estimated total fat (N = 4015)	1.000	REF	‐	1.000	REF	‐	1.000	REF	‐
Low estimated total MUFA^b^ & high estimated total fat (*N* = 460)	1.455	1.033–2.043	**0.031**	1.224	0.717–2.012	0.440	1.464	0.917–2.280	0.100
High estimated total MUFA^b^ & low estimated total fat (*N* = 171)	1.180	0.684–2.004	0.545	1.320	0.590–2.702	0.471	1.405	0.698–2.684	0.319
High estimated total MUFA & high estimated total fat (*N* = 4440)	1.214	1.037–1.423	0.016*	1.167	0.925–1.474	0.192	1.201	0.968–1.490	0.096
iv) Linoleic acids (LAs)									
Low estimated total LA & low estimated total fat (*N* = 3853)	1.000	REF	‐	1.000	REF	‐	1.000	REF	‐
Low estimated total LA^b^ & high estimated total fat (*N* = 622)	1.579	1.176–2.118	0.003*	1.661	1.090–2.490	**0.015**	1.462	0.970–2.168	0.064
High estimated total LA^b^ & low estimated total fat (*N* = 408)	0.967	0.656–1.410	0.863	0.936	0.508–1.629	0.823	1.096	0.648–1.787	0.723
High estimated total LA & high estimated total fat (*N* = 4203)	1.198	1.019–1.409	**0.028**	1.175	0.926–1.491	0.185	1.218	0.977–1.519	0.080
v) Alpha‐linolenic acids (ALAs)									
Low estimated total ALA & low estimated total fat (*N* = 3753)	1.000	REF	‐	1.000	REF	‐	1.000	REF	‐
Low estimated total ALA^b^ & high estimated total fat (*N* = 726)	1.067	0.803–1.413	0.652	1.027	0.669–1.544	0.899	1.130	0.764–1.646	0.510
High estimated total ALA^b^ & low estimated total fat (*N* = 798)	1.171	0.879–1.555	0.278	1.167	0.757–1.759	0.472	1.101	0.730–1.629	0.639
High estimated total ALA & high estimated total fat (*N* = 3813)	1.178	0.996–1.393	0.056	1.185	0.928–1.513	0.174	1.217	0.968–1.531	0.093

*Note*: All disease cases were compared to the non‐atopic non‐AD controls which were subjects with negative skin prick test response to two common house dust mites and absence of a recurrent itchy rash in flexural areas.

^a^ Multivariable logistic regression analysis was controlled for potential confounding effects from age, gender, BMI, parental eczema, use of alcohol, engagement in physical activities, use of TV/computer, and parental education.

^b^ For these categories, the category of moderate estimated total fatty acids was combined with the respective estimated fatty acids category to obtain a larger sample size that improved statistical empowerment for this analysis.

^c^ *P*‐value obtained from logistic regression has been adjusted by False discovery rate (FDR) correction. *P* < 0.05 was considered statistically significant. Unadjusted *p* < 0.05 was bolded and adjusted *p* < 0.05 was further indicated with an asterisk.

### 3.4 Fatty acids in diets do not interact to influence AD aggravation

As TFAs and SFAs are rarely consumed in isolation and most foods consist of a variety type of FAs, a SF analysis was conducted to determine if TFAs and/or SFAs interact with other FAs in diets to enhance the disease susceptibility (Table S3). Our results revealed that there were no significant synergism (greater than additive effect) or antagonism (lesser than additive effect) between any FAs combination to influence AD disease exacerbation. However, a high total estimated TFAs and SFAs in combination with other FAs in diets pronounced the associated odds of exacerbated AD.

## 4 DISCUSSION

Though excessive dietary fats in general are hypothesised to be potentially proinflammatory and can positively influence the T‐helper 2 cells' immune responses, the fundamental pathomechanism of how dietary fats and their subtypes act on the immune system cause allergic diseases remains poorly understood.^30^, ^31^, ^32^ Furthermore, large epidemiological studies focussing on different FAs and AD exacerbations are lacking. Therefore, our study aimed to understand through an epidemiological lens in providing a valuable starting point of how frequent intake for various FAs in diets improves or worsen AD symptoms. Here, we highlighted a positive relationship between AD and a high intake of TFAs and SFAs in diets that was independent of the total fat intake.

Processed foods with high TFAs including fast foods and margarine have been associated with risks of AD in children and adolescents.^7^, ^33^ In this study focussing on young Chinese adults, we also observed a dose‐dependent increase in the associated risk of AD exacerbations with a higher dietary TFAs consumption. Evidence indicates that diets high in TFAs are implicated in the exacerbation of inflammation and immune dysfunction which are key features of AD.^34^ TFAs intake was positively related to plasma inflammatory biomarkers, such as C‐reactive protein (CRP), interleukin‐6 (IL‐6), and E‐selectin.^35^ These pro‐inflammatory biomarkers were elevated in the serum of AD patients, and particularly, E‐selectin was proposed to be a useful biomarker for AD disease activity.^36^, ^37^, ^38^ As TFAs are commonly found in commercial deep‐fried foods and pastries in diets, the Singapore Health Promotion Board advocates trans‐fat‐free food choices (<0.50 g/100g) among individuals in reducing the risk of chronic inflammatory diseases.^39^

Compared to TFAs, SFAs are found more commonly in a variety of foods, particularly in butter, eggs, meat products, and pastries. The National Nutrition Survey in Singapore has highlighted stir‐fried vegetables, meat dishes containing coconut milk, and fried noodles to contribute to high SFAs in the diet of adults.^28^ In our study, high SFAs in diets showed strong positive associations with AD exacerbations, supporting past studies that underscored their links with inflammation and AD. A birth‐cohort study found that long‐chain (LC)‐SFAs in maternal breastmilk were regarded as a damage‐associated molecular pattern that triggered inflammatory cascades involving type 3 innate lymphoid cells (ILC3s). It was further revealed that breastfed Rag1 knockout mice model displayed AD onset later in life, suggesting LC‐SFAs played a role in worsening AD during early life by upregulating ILC3s and their associated proinflammatory cytokines such as IL‐17 and IL‐22.^12^ Moreover, SFAs have been suggested to mimic lipopolysaccharide to trigger TLR4 signalling in inducing an SFA‐inflammation interaction.^40^ Therefore, it is important for clinicians and dieticians to inform their patients, especially those with an atopy background and/or history of AD symptoms, about their personal dietary choices for lesser SFAs.

Dysregulated D6D has gained interest as a novel hallmark of AD, and genetics studies reported AD patients to have a lower expression of the Fatty Acid Desaturase 2 gene, which encodes for D6D.^41^, ^42^ Impairment of D6D leads to slower metabolism of LAs to gamma‐linolenic acids (GLAs) and altered human polyunsaturated fatty acids (PUFAs) metabolism.^43^, ^44^, ^45^ Young AD adults with dysregulated D6D showed elevated cis‐LAs and reduced GLAs in their plasma phospholipids.^46^ The conversion of certain SFAs such as palmitic acid to *cis*‐6‐hexadecenoic acid (C16:1Δ6) is primarily regulated by D6D. AD patients with deficit C16:1Δ6 have a higher susceptibility of skin colonisation by *Staphylococcus aureus*.^47^ Thus, it may be worthwhile to explore the activity levels of D6D in the regulation of FAs metabolism for intervention trials involving FAs. Although it was not fully understood, FAs' metabolism was implicated in the differentiation and survivability of T Lymphocytes to affect host immune system.^48^ Our epidemiological findings, while preliminary, may support the notion that excessive FAs in diets shape immune responses to encourage the development and even exacerbation of inflammatory diseases like AD.

Although previous studies have suggested that gender can influence fatty acid consumption,^49^, ^50^ our findings did not support this notion. The association between fatty acids intake in diets and AD remained significant and independent of gender. This suggests that the impact of dietary fatty acids on the associated risk of AD is consistent across both men and women in our study population. Diets rich in industrial TFAs result in an imbalance in the composition of the gut microbiota by promoting the proliferation of *Desulfovibrionaceae* and *Proteobacteria* while inhibiting the growth of *Bacteriodetes*.^51^ GI bacteria such as *Bifidobacterium*, *Lactobacillus*, and *Roseburia* spp. were shown to regulate PUFA metabolism and in turn, regulate histamine release.^52^ Hence, this evidence suggests GI microbiome dysbiosis may play a critical role in immune regulation while dietary fatty acids can influence the normal homoeostasis of GI microbiome and AD development.

Despite the possibility of recall bias, our large sample size minimises the impact of individual measurement errors and enhances the study's statistical power to detect associations between dietary fatty acid intake and AD. The use of a validated semi‐quantitative food frequency questionnaire (FFQ) administered by trained investigators further reduces any unnecessary misunderstandings in dietary reporting. Being a cross‐sectional study, causality between frequent intakes of various FAs and AD exacerbations cannot be necessarily established now. However, standardized methods and validated guidelines for the data collection on AD and dietary habits were adopted which improved the reliability and comparability of our association findings. Moreover, we have controlled for confounding variables such as lifestyles, genetics, socioeconomic, anthropometry, age, and gender that could impact the association between FAs and AD. Another limitation is that we only studied five main types of dietary FAs by referencing the USDA database and it might not fully capture the other subtypes of FAs consumed in diets. We seek to replicate these findings independently by cross‐validating with other cohorts using a detailed FFQ that surveys an exhaustive list of foods with known nutritional FAs content. A randomised controlled feeding trial involving a restricted diet with reduced fats, particularly SFAs and TFAs among AD patients with severe symptoms should be carefully planned to demonstrate the protective effects of lower FAs intake on AD exacerbations.

Ultimately, we hope that our findings provide valuable insights into the potential role of dietary factors in terms of FAs consumption in the development and severity of AD. These insights may inform public health policies and dietary recommendations, as well as guide future research on the pathomechanisms underlying an association between intake frequencies of FAs and AD exacerbations.

## CONFLICT OF INTEREST STATEMENT

F.T.C. reports grants from National University of Singapore, Singapore Ministry of Education Academic Research Fund, Singapore Immunology Network, National Medical Research Council (NMRC) (Singapore), Biomedical Research Council (BMRC) (Singapore), National Research Foundation (NRF) (Singapore), Singapore Food Agency (SFA), and the Agency for Science Technology and Research (A*STAR) (Singapore), during the conduct of the study; and consultancy fees from Sime Darby Technology Centre; First Resources Ltd; Genting Plantation, Olam International, and Syngenta Crop Protection, outside the submitted work.

## AUTHOR CONTRIBUTION

**Jun Jie Lim**: Data curation (lead); Formal analysis (lead); Investigation (lead); Project administration (equal); Resources (equal); Visualisation (equal); Writing – original draft (lead); Writing – review & editing (lead). **Sing Wei Lim**: Data curation (equal); Formal analysis (equal); Investigation (supporting); Project administration (equal); Software (equal); Writing – original draft (supporting). **Kavita Reginald**: Investigation (equal); Project administration (equal); Resources (equal). **Yee‐How Say**: Investigation (equal); Project administration (equal); Resources (equal). **Mei Hui Liu**: Investigation (equal); Project administration (equal); Resources (equal); Writing – review & editing (equal). **Fook Tim Chew**: Conceptualisation (lead); Funding acquisition (lead); Investigation (lead); Methodology (lead); Project administration (lead); Supervision (lead).

## ETHICS STATEMENT

This study was conducted in accordance with the principles of the Declaration of Helsinki and Good Clinical Practices, and in compliance with local regulatory requirements. Cross‐sectional studies in Singapore were conducted on the National University of Singapore (NUS) campus annually between 2005 and 2022, under the approval of the Institutional Review Board (NUS‐IRB Reference Code: NUS‐07‐023, NUS‐09‐256, NUS‐10‐445, NUS‐13‐075, NUS‐14‐150, and NUS‐18‐036) and by the Helsinki declaration. Cross‐sectional studies in Malaysia were held in the Universiti Tunku Abdul Rahman (UTAR), and Sunway University. Ethical approval was granted respectively from the Scientific and Ethical Review Committee (SERC) of UTAR (Ref. code: U/SERC/03/2016) and Sunway University Research Ethics Committee (Ref. code: SUREC 2019/029). Before the data collection, all participants involved signed an informed consent form.

## Supporting information

Table S1

Table S2

Table S3

## Data Availability

The data underlying this article will be shared on reasonable request to the corresponding author (F.T.C.).
