# Supplementary material for: Association of frequent intake of trans fatty acids and saturated fatty acids in diets with increased susceptibility of atopic dermatitis exacerbation in young Chinese adults: A cross‐sectional study in Singapore/Malaysia
Source: Skin Health Dis. 2024 Jun 26;4(4):e330. doi: 10.1002/ski2.330 (PMC11297457; doi:10.1002/ski2.330)
Supplement: Supplementary file 1 — Table S1 [file SKI2-4-e330-s003.docx]

**Supplemental Table 1.** Nutritional Information on the total average estimated fatty acids amount in a weekly diet for each food type asked in the questionnaire. Data was retrieved from the United States Department of Agriculture (USDA) database. (A) Total average estimated fatty acids amount for each food type. (**B**) The list of specific foods used to compute the total average estimated fatty acids amount for each food type.

| **(A)Food Types** | **Estimated Average Amount of dietary fatty acids per 100 unit serving (g)** | | | | | |
| --- | --- | --- | --- | --- | --- | --- |
|  | **LAs** | **ALAs** | **TFAs** | **SFAs** | **MUFAs** | **Total fat** |
| Butter | 2.73 | 0.315 | 3.28 | 51.0 | 21.7 | 81.1 |
| Margarine | 17.9 | 2.67 | 6.86 | 12.7 | 27.7 | 65.2 |
| Nuts | 10.6 | 0.131 | 0.0350 | 7.04 | 34.7 | 54.4 |
| Meat^1^ | 1.25 | 0.120 | 0.514 | 6.43 | 7.64 | 17.4 |
| Burgers/fast foods | 4.63 | 0.410 | 0.180 | 3.93 | 5.77 | 16.1 |
| Eggs | 1.06 | 0.180 | 0.0380 | 3.40 | 4.89 | 11.6 |
| Cereals^2^ | 1.25 | 0.120 | 0.0210 | 0.113 | 0.580 | 3.38 |
| Seafood^3^ | 0.736 | 0.0477 | 0.00350 | 0.717 | 1.07 | 3.22 |
| Milk | 0.280 | 0.0620 | 0.185 | 1.50 | 0.830 | 2.84 |
| Pasta | 0.420 | 0.0340 | 0.0404 | 0.380 | 0.409 | 1.57 |
| Pulses^4^ | 0.105 | 0.0975 | 0.00 | 0.0742 | 0.0520 | 0.435 |
| Rice | 0.132 | 0.00870 | 0.00 | 0.0870 | 0.145 | 0.410 |
| Fruits | 0.0478 | 0.0167 | 0.00 | 0.0540 | 0.0480 | 0.225 |
| Vegetables^5^ | 0.0457 | 0.0310 | 0.00 | 0.0359 | 0.0190 | 0.191 |
| Potatoes | 0.0520 | 0.00650 | 0.00 | 0.0325 | 0.00150 | 0.135 |
| Probiotic drinks^6^ | 0.00 | 0.00 | 0.00 | 0.00 | 0.00 | 0.00 |

Abbreviations: LA: Linoleic acid; ALA: Alpha-linolenic fatty acid; TFA: Trans fatty acid; SFA: Saturated fatty acid; MUFA: Monounsaturated fatty acid.

All values are in grams/serving and are taken from an average of FAs amount in a representative type of foods within each category. The information on nutrient amount was obtained from the United States Department of Agriculture (USDA) Food Search Database under https://fdc.nal.usda.gov/, at last accessed on 18 August 2022.

^1^ For example, beef, lamb, chicken, pork. ^2^ Including bread. ^3^ Including fish. ^4^ Peas, beans, lentils. ^5^ Green and root. ^6^ Reflected as Yakult/Vitagen/similar yoghurt drinks in the questionnaire. Yakult and Vitagen are common commercial cultured probiotic drinks in Singapore and Malaysia.

| 1. **List of Foods^a^** | **Estimated Amount of dietary fatty acids per 100 unit serving (g)** | | | | | |
| --- | --- | --- | --- | --- | --- | --- |
|  | **LAs** | **ALAs** | **TFAs** | **SFAs** | **MUFAs** | **Total Fats** |
| - **Butter** | | | | | | |
| Butter, without salt | 2.73 | 0.315 | NA | 50.5 | 21 | 81.1 |
| Butter, salted | 2.73 | 0.315 | 3.28 | 51.4 | 22.32 | 81.1 |
| - **Margarine** | | | | | | |
| Margarine, regular, hard, soybean (hydrogenated) | 19.4 | 1.5 | NA | 16.7 | 39.3 | 80.5 |
| Margarine spread, 35-39% fat, tub | 13.8 | 2.29 | 0.079 | 8.66 | 11.2 | 38 |
| Margarine, margarine-type vegetable oil spread, 70% fat, soybean and partially hydrogenated soybean, spread | 18.1 | 2.13 | 14.8 | 13.6 | 33.5 | 70.2 |
| Margarine-like, margarine-butter blend, soybean oil and butter | 21.5 | 2.64 | 15.0 | 14.2 | 30.3 | 80.3 |
| Margarine, regular, 80% fat,composite, tub, with salt | 21.7 | 5.03 | 5.83 | 14.2 | 36.4 | 80.2 |
| Margarine-like spread, BENECOL Light Spread | 9.72 | 2.19 | 2.21 | 4.77 | 18.9 | 38.7 |
| Margarine, Margarine-like vegetable oil spread, 67-70% fat, tub | 20.8 | 2.94 | 3.26 | 16.7 | 24.6 | 68.3 |
| - **Nuts** | | | | | | |
| Nuts, almonds | 18.2 | 0.003 | 0.015 | 3.8 | 31.6 | 49.9 |
| Nuts, macadamia nuts, raw | 1.3 | 0.206 | NA | 12.1 | 58.9 | 75.8 |
| Nuts, hazelnuts or filberts | 7.83 | 0.087 | NA | 4.46 | 45.7 | 60.8 |
| Nuts, cashew nuts, raw | 7.78 | 0.062 | NA | 7.78 | 23.8 | 43.8 |
| Nuts, pistachio nuts, raw | 14.1 | 0.289 | 0 | 5.91 | 23.3 | 45.3 |
| Nuts, mixed nuts, oil roasted, with peanuts, with salt added | 14.5 | 0.082 | 0.055 | 8.71 | 28.5 | 54 |
| Nuts, mixed nuts, dry roasted, with peanuts, with salt added | 10.5 | 0.19 | NA | 6.5 | 31.4 | 51.4 |
| - **Burgers/fast food** | | | | | | |
| Fast foods, potato, French fried in vegetable oil | 4.95 | 0.436 | 0.06 | 2.34 | 5.97 | 14.7 |
| Fast foods, hamburger; single, large patty; with condiments, vegetables, and mayonnaise | 2.83 | 0.275 | 0.436 | 3.97 | 4.67 | 12.4 |
| Fast foods, onion rings, breaded and fried | 11.5 | 0.619 | 0.164 | 4.19 | 6.88 | 25.2 |
| Fast foods, cheeseburger; single, large patty; with condiments | 1.01 | 0.127 | NA | 7.03 | 5.96 | 14.4 |
| McDONALD’S, McCHICKEN Sandwich | 4.45 | 0.447 | 0.045 | 2.35 | 4.4 | 13.2 |
| Fast Foods, Fried Chicken, Thigh, meat and skin and breading | 2.47 | 0.329 | 0.252 | 4.83 | 8.48 | 18.1 |
| McDONALD’S, FILET-O-FISH | 5.19 | 0.639 | 0.128 | 2.83 | 4 | 14.6 |
| - **Eggs** | | | | | | |
| Eggs, grade A, large, whole | 1.46 | 0.011 | NA | 3.2 | 3.63 | 9.96 |
| Egg, whole, raw, fresh | 1.56 | 0.048 | 0.038 | 3.13 | 3.66 | 9.51 |
| Egg, duck, whole, fresh, raw | 0.558 | 0.102 | NA | 3.68 | 6.52 | 13.8 |
| Egg, goose, whole, fresh, raw | 0.68 | 0.554 | NA | 3.6 | 5.75 | 13.3 |
| - **Meat (including beef, lamb, chicken, pork)** | | | | | | |
| Beef, ground, 70% lean meat / 30% fat, raw | 0.577 | 0.084 | 1.82 | 11.8 | 14.2 | 30 |
| Lamb, ground, raw | 1.36 | 0.42 | NA | 10.2 | 9.6 | 23.4 |
| Pork, ground, 84% lean / 16% fat, raw | 1.84 | 0.089 | 0.124 | 4.93 | 6.68 | 16 |
| Chicken, ground, raw | 1.32 | 0.071 | 0.065 | 2.3 | 3.61 | 8.1 |
| Chicken, broilers or fryers, drumstick, meat and skin, raw | 1.7 | 0.088 | 0.047 | 2.46 | 3.72 | 9.2 |
| Beef, ground, unspecified fat content, cooked | 0.34 | 0.049 | NA | 5.6 | 6.4 | 14.5 |
| Pork, fresh, ground, cooked | 1.64 | 0.07 | NA | 7.72 | 9.25 | 20.8 |
| - **Milk** | | | | | | |
| Milk, chocolate, low fat, reduced sugar | 0.045 | 0.005 | 0.036 | 0.616 | 0.29 | 1.04 |
| Milk, canned, condensed, sweetened | 0.216 | 0.121 | NA | 5.49 | 2.43 | 8.7 |
| Milk, whole, 3.25% milkfat, without added vitamin A and vitamin D | 0.12 | 0.075 | NA | 1.86 | 0.812 | 3.27 |
| Soy milk, unsweetened, plain, shelf stable | 0.988 | 0.166 | 0.002 | 0.314 | 0.416 | 1.88 |
| Milk, low-fat, fluid, 1% milkfat, with added vitamin A and vitamin D | 0.03 | 0.004 | 0.036 | 0.633 | 0.277 | 0.95 |
| Almond milk, unsweetened, plain, shelf stable | 0.276 | 0.001 | 0 | 0.104 | 0.729 | 1.22 |
| - **Pasta** | | | | | | |
| Pasta, cooked, enriched, without added salt | 0.295 | 0.024 | 0 | 0.176 | 0.131 | 0.93 |
| Pasta, homemade, made with egg, cooked | 0.459 | 0.045 | NA | 0.408 | 0.508 | 1.74 |
| Pasta mix, classic beef, unprepared | 0.768 | 0.039 | 0.106 | 0.439 | 0.501 | 1.76 |
| Pasta mix, Italian lasagna, unprepared | 0.388 | 0.027 | 0.089 | 0.443 | 0.423 | 1.97 |
| Pasta with tomato sauce, no meat, canned | 0.19 | 0.015 | 0.007 | 0.167 | 0.109 | 0.71 |
| Pasta with Sliced Franks in Tomato Sauce, canned entrée | 0.277 | 0.051 | NA | 0.791 | 1.02 | 2.38 |
| Pasta, whole grain, 51% whole wheat, remaining unenriched semolina, cooked | 0.545 | 0.039 | 0 | 0.239 | 0.171 | 1.5 |
| - **Seafood (including fish)** | | | | | | |
| Mollusks, scallops, mixed species, raw | 0.008 | 0.003 | 0.005 | 0.128 | 0.048 | 0.49 |
| Fish, whitefish, mixed species, cooked, dry heat | 0.349 | 0.235 | NA | 1.16 | 2.56 | 7.51 |
| Crustaceans, crab, blue, raw | 0.012 | 0 | NA | 0.222 | 0.192 | 1.08 |
| Mollusks, cuttlefish, mixed species, cooked, moist heat | 0.004 | 0.002 | NA | 0.236 | 0.162 | 1.4 |
| Crustaceans, shrimp, cooked | 0.018 | 0.001 | 0.002 | 0.056 | 0.048 | 0.28 |
| Fish, salmon, chinook, raw | 0.122 | 0.089 | NA | 3.1 | 4.4 | 10.4 |
| Molluscs, squid, mixed species, raw | 0.002 | 0.004 | NA | 0.358 | 0.107 | 1.38 |
| - **Cereals (including bread)** | | | | | | |
| Cereals ready-to-eat, POST, Honeycomb Cereal | 0.875 | 0.035 | 0.01 | 1.29 | 0.6 | 2.93 |
| Bread, wheat | 1.45 | 0.161 | 0.033 | 0.697 | 0.612 | 4.53 |
| Bread, French or Vienna (includes sourdough) | 0.79 | 0.064 | 0.005 | 0.529 | 0.362 | 2.42 |
| Bread, white, commercially prepared | 1.67 | 0.2 | 0.035 | 0.821 | 0.717 | 3.45 |
| Bread, whole wheat, commercially prepared | 1.46 | 0.141 | 0.022 | 0.732 | 0.616 | 3.55 |
| - **Rice** | | | | | | |
| Rice, white, medium grain, cooked, unenriched | 0.046 | 0.01 | NA | 0.057 | 0.065 | 0.21 |
| Rice, white, glutinous, unenriched, cooked | 0.066 | 0.003 | NA | 0.039 | 0.07 | 0.19 |
| Rice, brown, medium grain, cooked (Includes foods for USDA’s Food Distribution Program) | 0.283 | 0.013 | NA | 0.165 | 0.3 | 0.83 |
| - **Potatoes** | | | | | | |
| Sweet potato, cooked, boiled, without skin | 0.061 | 0 | 0 | 0.031 | 0 | 0.14 |
| Potatoes, baked, flesh and skin, with salt | 0.043 | 0.013 | 0 | 0.034 | 0.003 | 0.13 |
| - **Pulses (Peas, beans, lentils)** | | | | | | |
| Mung beans, mature seeds, cooked, boiled, without salt | 0.119 | 0.009 | 0 | 0.116 | 0.054 | 0.38 |
| Beans, French, mature seeds, cooked, boiled, without salt | 0.166 | 0.287 | 0 | 0.083 | 0.052 | 0.76 |
| Beans, snap, green, raw | 0.044 | 0.069 | 0 | 0.05 | 0.01 | 0.22 |
| Lentils, mature seeds, cooked, boiled, with salt | 0.137 | 0.037 | 0 | 0.053 | 0.064 | 0.38 |
| Beans, kidney, red, mature seeds, cooked, boiled, without salt | 0.107 | 0.168 | 0 | 0.072 | 0.039 | 0.5 |
| Beans, baked, canned, plain, or vegetarian | 0.055 | 0.015 | 0 | 0.071 | 0.095 | 0.37 |
| - **Vegetables (greens and roots)** | | | | | | |
| Spinach, raw | 0.026 | 0.138 | 0 | 0.063 | 0.01 | 0.39 |
| Lettuce, green leaf, raw | 0.024 | 0.058 | 0 | 0.02 | 0.006 | 0.15 |
| Carrots, cooked, boiled, drained, without salt | 0.087 | 0.001 | 0 | 0.03 | 0.006 | 0.18 |
| Cucumber, with peel, raw | 0.028 | 0.005 | 0 | 0.037 | 0.005 | 0.11 |
| Cabbage, raw | 0.017 | 0 | 0 | 0.034 | 0.017 | 0.1 |
| Tomatoes, red, ripe, raw, year-round average | 0.08 | 0.003 | 0 | 0.028 | 0.031 | 0.2 |
| Mung beans, mature seeds, sprouted, cooked, stir-fried | 0.058 | 0.011 | 0 | 0.039 | 0.056 | 0.21 |
| - **Fruits** | | | | | | |
| Bananas, raw | 0.046 | 0.027 | 0 | 0.112 | 0.032 | 0.33 |
| Watermelon, raw | 0.05 | 0 | 0 | 0.016 | 0.037 | 0.15 |
| Apples, raw, with skin (Includes foods for USDA’s Food Distribution Program) | 0.043 | 0.009 | 0 | 0.058 | 0.007 | 0.17 |
| Oranges, raw, with peel | 0.044 | 0.016 | 0 | 0.035 | 0.055 | 0.3 |
| Papayas, raw | 0.011 | 0.047 | 0 | 0.081 | 0.072 | 0.26 |
| Pears, raw | 0.093 | 0.001 | 0 | 0.022 | 0.084 | 0.14 |
| - **Probiotic Drinks** | | | | | | |
| Yakult probiotic drink | 0 | 0 | 0 | 0 | 0 | 0 |
| Yakult LIGHT probiotic drink | 0 | 0 | 0 | 0 | 0 | 0 |
| ^a^ No available information on the USDA is marked with NA. | | | | | | |
