# Supplementary material for: Association of frequent intake of trans fatty acids and saturated fatty acids in diets with increased susceptibility of atopic dermatitis exacerbation in young Chinese adults: A cross‐sectional study in Singapore/Malaysia
Source: Skin Health Dis. 2024 Jun 26;4(4):e330. doi: 10.1002/ski2.330 (PMC11297457; doi:10.1002/ski2.330)
Supplement: Supplementary file 2 — Table S2 [file SKI2-4-e330-s002.docx]

**Supplemental Table 2.** Information on the various dietary fatty acids indices based on the preliminary 13,561 young Chinese adults from the Singapore/Malaysia Cross-sectional Genetics Epidemiology Study (SMCGES) cohort. Dietary indices were formulated based on the intake frequencies of 16 food types and their usual portion size of 100g/serving as estimated from the United States Department of Agriculture (USDA) nutrient database. Values are shown in grams/serving.

| g/week | **Dietary Fat Indices** | | | | | |
| --- | --- | --- | --- | --- | --- | --- |
|  | **Total Fat** | **TFAs** | **SFAs** | **MUFAs** | **LAs** | **ALAs** |
| - **Cut-offs** | | | | | | |
| Low Estimated Total Amount (33^rd^ percentile cut-off) | ≤333.633 | ≤5.313 | ≤95.6977 | ≤141.7369 | ≤43.7298 | ≤4.0422 |
| Moderate Estimated Total Amount | Between 333.633 & 605.333 | Between 5.313  & 24.80252 | Between 95.6977  & 213.2327 | Between 142.0998 & 247.97 | Between 43.7298  & 87.8291 | Between 4.0422 & 9.4273 |
| High Estimated Total Amount (66^th^ percentile cut-off) | ≥605.333 | ≥24.8052 | ≥213.2327 | ≥247.97 | ≥87.8291 | ≥9.4273 |
| - **Range, Mean, Median, Standard Deviation** | | | | | | |
| Minimum | 0.0 | 0.0 | 0.0 | 0.0 | 0.0 | 0.0 |
| Maximum | 1807.4 | 78.098 | 617.94 | 738.9 | 288.67 | 29.751 |
| Mean | 539.1 | 18.976 | 191.21 | 216.9 | 75.72 | 7.993 |
| Median | 484.5 | 12.041 | 183.16 | 188.2 | 62.95 | 5.701 |
| SD | 357.2816 | 18.91647 | 139.4311 | 142.2801 | 55.97261 | 6.349385 |

Abbreviation: Trans fatty acids (TFAs); Saturated fatty acids (SFAs); Monounsaturated fatty acids (MUFAs); Linoleic acids (LAs); Alpha-linolenic fatty acids (ALAs).
