# Supplementary material for: Association of frequent intake of trans fatty acids and saturated fatty acids in diets with increased susceptibility of atopic dermatitis exacerbation in young Chinese adults: A cross‐sectional study in Singapore/Malaysia
Source: Skin Health Dis. 2024 Jun 26;4(4):e330. doi: 10.1002/ski2.330 (PMC11297457; doi:10.1002/ski2.330)
Supplement: Supplementary file 3 — Table S3 [file SKI2-4-e330-s001.docx]

**Supplemental Table 3.** Synergy Factor (SF) analysis for the interactions between the intake frequencies of 5 fatty acids (FAs) in diets to influence the associated risk for (a) atopic dermatitis (AD), (b) chronic AD, and (c) moderate-to-severe AD. Intake frequencies of FAs were examined, and these included trans fatty acids (TFAs), saturated fatty acids (SFAs), monounsaturated fatty acids (MUFAs), linoleic acids (LAs), and alpha-linolenic acids (ALAs). A SF p value < 0.05 signifies a statistically significance interaction between FAs.

| 1. **AD Presentation (Non-atopic non-AD controls vs. Atopic AD cases)** | | | | | | | |
| --- | --- | --- | --- | --- | --- | --- | --- |
| 1. **Interactions Between Trans Fatty Acids (TFAs) and Saturated Fatty Acids (SFAs)** | | | | | | | |
| TFA | SFA | Non-atopic Non-AD Controls | AD Cases | Odds Ratio | SF P Value | SF  (95% CI) | Interaction |
| - | - | 1140 | 662 | Reference | 0.951 | 1.013  (0.668-1.536) | NA |
| + | - | 126 | 76 | 1.039 |  |  |  |
| - | + | 113 | 96 | 1.463 |  |  |  |
| + | + | 906 | 810 | 1.539 |  |  |  |
| 1. **Interactions Between Trans Fatty Acids (TFAs) and Monounsaturated Fatty Acids (MUFAs)** | | | | | | | |
| TFA | MUFA | Non-atopic Non-AD Controls | AD Cases | Odds Ratio | SF P Value | SF  (95% CI) | Interaction |
| - | - | 954 | 568 | Reference | 0.591 | 0.913  (0.656-1.271) | NA |
| + | - | 312 | 170 | 0.915 |  |  |  |
| - | + | 139 | 144 | 1.740 |  |  |  |
| + | + | 880 | 762 | 1.454 |  |  |  |
| 1. **Interactions Between Trans Fatty Acids (TFAs) and Linoleic Acids (LAs)** | | | | | | | |
| TFA | LA | Non-atopic Non-AD Controls | AD Cases | Odds Ratio | SF P Value | SF  (95% CI) | Interaction |
| - | - | 939 | 561 | Reference | 0.446 | 0.866  (0.649-1.209) | NA |
| + | - | 327 | 177 | 0.906 |  |  |  |
| - | + | 176 | 187 | 1.778 |  |  |  |
| + | + | 843 | 719 | 1.427 |  |  |  |
| 1. **Interactions Between Trans Fatty Acids (TFAs) and Alpha-linolenic** **Acids (ALAs)** | | | | | | | |
| TFA | ALA | Non-atopic Non-AD Controls | AD Cases | Odds Ratio | SF P Value | SF  (95% CI) | Interaction |
| - | - | 1053 | 614 | Reference | 0.839 | 0.964  (0.679-1.369) | NA |
| + | - | 213 | 124 | 0.998 |  |  |  |
| - | + | 147 | 135 | 1.575 |  |  |  |
| + | + | 872 | 771 | 1.516 |  |  |  |
| 1. **Interactions Between Saturated Fatty Acids (SFAs) and Monounsaturated Fatty Acids (MUFAs)** | | | | | | | |
| SFA | MUFA | Non-atopic Non-AD Controls | AD Cases | Odds Ratio | SF P Value | SF  (95% CI) | Interaction |
| - | - | 1036 | 621 | Reference | 0.624 | 0.912  (0.632-1.317) | NA |
| + | - | 217 | 118 | 0.907 |  |  |  |
| - | + | 114 | 120 | 1.756 |  |  |  |
| + | + | 899 | 783 | 1.453 |  |  |  |
| 1. **Interactions Between Saturated Fatty Acids (SFAs) and Linoleic Acids (LAs)** | | | | | | | |
| SFA | LA | Non-atopic Non-AD Controls | AD Cases | Odds Ratio | SF P Value | SF  (95% CI) | Interaction |
| - | - | 1004 | 596 | Reference | 0.147 | 0.784  (0.565-1.089) | NA |
| + | - | 249 | 143 | 0.967 |  |  |  |
| - | + | 160 | 179 | 1.885 |  |  |  |
| + | + | 853 | 724 | 1.430 |  |  |  |
| 1. **Interactions Between Saturated Fatty Acids (SFAs) and Alpha-linolenic** **Acids (ALAs)** | | | | | | | |
| SFA | ALA | Non-atopic Non-AD Controls | AD Cases | Odds Ratio | SF P Value | SF  (95% CI) | Interaction |
| - | - | 1052 | 623 | Reference | 0.427 | 0.867  (0.611-1.232) | NA |
| + | - | 201 | 116 | 0.975 |  |  |  |
| - | + | 148 | 152 | 1.734 |  |  |  |
| + | + | 865 | 751 | 1.466 |  |  |  |
| 1. **Interactions Between Monounsaturated Fatty Acids (MUFAs) and Linoleic Acids (LAs)** | | | | | | | |
| MUFA | LA | Non-atopic Non-AD Controls | AD Cases | Odds Ratio | SF P Value | SF  (95% CI) | Interaction |
| - | - | 1117 | 701 | Reference | 0.021 | 0.575  (0.360-0.919) | Synergism |
| + | - | 96 | 76 | 1.261 |  |  |  |
| - | + | 64 | 74 | 1.842 |  |  |  |
| + | + | 974 | 817 | 1.337 |  |  |  |
| 1. **Interactions Between Monounsaturated Fatty Acids (MUFAs) and Alpha-linolenic** **Acids (ALAs)** | | | | | | | |
| MUFA | ALA | Non-atopic Non-AD Controls | AD Cases | Odds Ratio | SF P Value | SF  (95% CI) | Interaction |
| - | - | 974 | 621 | Reference | 0.340 | 0.856  (0.623-1.177) | NA |
| + | - | 239 | 156 | 1.024 |  |  |  |
| - | + | 195 | 186 | 1.496 |  |  |  |
| + | + | 843 | 705 | 1.312 |  |  |  |
| 1. **Interactions Between Linoleic Acids (LAs) and Alpha-linolenic** **Acids (ALAs)** | | | | | | | |
| LA | ALA | Non-atopic Non-AD Controls | AD Cases | Odds Ratio | SF P Value | SF  (95% CI) | Interaction |
| - | - | 978 | 634 | Reference | 0.984 | 1.004  (0.715-1.408) | NA |
| + | - | 228 | 147 | 0.995 |  |  |  |
| - | + | 163 | 133 | 1.259 |  |  |  |
| + | + | 905 | 737 | 1.256 |  |  |  |

| 1. **AD Chronicity (Non-atopic non-AD controls vs. chronic AD)** | | | | | | | |
| --- | --- | --- | --- | --- | --- | --- | --- |
| 1. **Interactions Between Trans Fatty Acids (TFAs) and Saturated Fatty Acids (SFAs)** | | | | | | | |
| TFA | SFA | Non-atopic Non-AD Controls | Chronic AD | Odds Ratio | SF P Value | SF  (95% CI) | Interaction |
| - | - | 1140 | 231 | Reference | 0.882 | 1.048  (0.565-1.943) | NA |
| + | - | 126 | 25 | 0.979 |  |  |  |
| - | + | 113 | 31 | 1.354 |  |  |  |
| + | + | 906 | 255 | 1.389 |  |  |  |
| 1. **Interactions Between Trans Fatty Acids (TFAs) and Monounsaturated Fatty Acids (MUFAs)** | | | | | | | |
| TFA | MUFA | Non-atopic Non-AD Controls | Chronic AD | Odds Ratio | SF P Value | SF  (95% CI) | Interaction |
| - | - | 954 | 194 | Reference | 0.421 | 0.822  (0.510-1.325) | NA |
| + | - | 312 | 62 | 0.977 |  |  |  |
| - | + | 139 | 47 | 1.663 |  |  |  |
| + | + | 880 | 239 | 1.336 |  |  |  |
| 1. **Interactions Between Trans Fatty Acids (TFAs) and Linoleic Acids (LAs)** | | | | | | | |
| TFA | LA | Non-atopic Non-AD Controls | Chronic AD | Odds Ratio | SF P Value | SF  (95% CI) | Interaction |
| - | - | 939 | 193 | Reference | 0.833 | 1.051  (0.660-1.674) | NA |
| + | - | 327 | 63 | 0.937 |  |  |  |
| - | + | 176 | 50 | 1.382 |  |  |  |
| + | + | 843 | 236 | 1.362 |  |  |  |
| 1. **Interactions Between Trans Fatty Acids (TFAs) and Alpha-linolenic** **Acids (ALAs)** | | | | | | | |
| TFA | ALA | Non-atopic Non-AD Controls | Chronic AD | Odds Ratio | SF P Value | SF  (95% CI) | Interaction |
| - | - | 1053 | 214 | Reference | 0.202 | 1.429  (0.826-2.473) | NA |
| + | - | 213 | 42 | 0.970 |  |  |  |
| - | + | 147 | 31 | 1.038 |  |  |  |
| + | + | 872 | 255 | 1.439 |  |  |  |
| 1. **Interactions Between Saturated Fatty Acids (SFAs) and Monounsaturated Fatty Acids (MUFAs)** | | | | | | | |
| SFA | MUFA | Non-atopic Non-AD Controls | Chronic AD | Odds Ratio | SF P Value | SF  (95% CI) | Interaction |
| - | - | 1036 | 210 | Reference | 0.108 | 0.656  (0.392-1.097) | NA |
| + | - | 217 | 45 | 1.023 |  |  |  |
| - | + | 114 | 45 | 1.947 |  |  |  |
| + | + | 899 | 238 | 1.306 |  |  |  |
| 1. **Interactions Between Saturated Fatty Acids (SFAs) and Linoleic Acids (LA)** | | | | | | | |
| SFA | LA | Non-atopic Non-AD Controls | Chronic AD | Odds Ratio | SF P Value | SF  (95% CI) | Interaction |
| - | - | 1004 | 202 | Reference | 0.282 | 0.769  (0.477-1.241) | NA |
| + | - | 249 | 53 | 1.058 |  |  |  |
| - | + | 160 | 53 | 1.646 |  |  |  |
| + | + | 853 | 230 | 1.340 |  |  |  |
| 1. **Interactions Between Saturated Fatty Acids (SFAs) and Alpha-linolenic** **Acids (ALAs)** | | | | | | | |
| SFA | ALA | Non-atopic Non-AD Controls | Chronic AD | Odds Ratio | SF P Value | SF  (95% CI) | Interaction |
| - | - | 1052 | 215 | Reference | 0.975 | 1.008  (0.597-1.703) | NA |
| + | - | 201 | 40 | 0.974 |  |  |  |
| - | + | 148 | 42 | 1.389 |  |  |  |
| + | + | 865 | 241 | 1.363 |  |  |  |
| 1. **Interactions Between Monounsaturated Fatty Acids (MUFAs) and Linoleic Acids (LAs)** | | | | | | | |
| MUFA | LA | Non-atopic Non-AD Controls | AD Cases | Odds Ratio | SF P Value | SF  (95% CI) | Interaction |
| - | - | 1117 | 227 | Reference | 0.914 | 1.045  (0.470-2.324) | NA |
| + | - | 96 | 33 | 1.691 |  |  |  |
| - | + | 64 | 10 | 0.769 |  |  |  |
| + | + | 974 | 269 | 1.359 |  |  |  |
| 1. **Interactions Between Monounsaturated Fatty Acids (MUFAs) and Alpha-linolenic** **Acids (ALAs)** | | | | | | | |
| MUFA | ALA | Non-atopic Non-AD Controls | AD Cases | Odds Ratio | SF P Value | SF  (95% CI) | Interaction |
| - | - | 974 | 208 | Reference | 0.486 | 1.185  (0.735-1.911) | NA |
| + | - | 239 | 52 | 1.019 |  |  |  |
| - | + | 195 | 50 | 1.201 |  |  |  |
| + | + | 843 | 261 | 1.450 |  |  |  |
| 1. **Interactions Between Linoleic Acids (LAs) and Alpha-linolenic** **Acids (ALAs)** | | | | | | | |
| LA | ALA | Non-atopic Non-AD Controls | AD Cases | Odds Ratio | SF P Value | SF  (95% CI) | Interaction |
| - | - | 978 | 217 | Reference | 0.660 | 1.121  (0.675-1.860) | NA |
| + | - | 228 | 52 | 1.028 |  |  |  |
| - | + | 163 | 38 | 1.051 |  |  |  |
| + | + | 905 | 243 | 1.210 |  |  |  |

| 1. **AD Severity (Non-atopic non-AD controls vs. Moderate-to-severe AD)** | | | | | | | |
| --- | --- | --- | --- | --- | --- | --- | --- |
| 1. **Interactions Between Trans Fatty Acids (TFAs) and Saturated Fatty Acids (SFAs)** | | | | | | | |
| TFA | SFA | Non-atopic Non-AD Controls | Moderate-to-severe AD | Odds Ratio | SF P Value | SF  (95% CI) | Interaction |
| - | - | 1140 | 248 | Reference | 0.463 | 1.256  (0.683-2.308) | NA |
| + | - | 126 | 25 | 0.912 |  |  |  |
| - | + | 113 | 33 | 1.342 |  |  |  |
| + | + | 906 | 303 | 1.537 |  |  |  |
| 1. **Interactions Between Trans Fatty Acids (TFAs) and Monounsaturated Fatty Acids (MUFAs)** | | | | | | | |
| TFA | MUFA | Non-atopic Non-AD Controls | Moderate-to-severe AD | Odds Ratio | SF P Value | SF  (95% CI) | Interaction |
| - | - | 954 | 215 | Reference | 0.635 | 1.122  (0.699-1.801) | NA |
| + | - | 312 | 58 | 0.825 |  |  |  |
| - | + | 139 | 49 | 1.564 |  |  |  |
| + | + | 880 | 287 | 1.447 |  |  |  |
| 1. **Interactions Between Trans Fatty Acids (TFAs) and Linoleic Acids (LAs)** | | | | | | | |
| TFA | LA | Non-atopic Non-AD Controls | Moderate-to-severe AD | Odds Ratio | SF P Value | SF  (95% CI) | Interaction |
| - | - | 939 | 211 | Reference | 0.824 | 1.052  (0.675-1.638) | NA |
| + | - | 327 | 62 | 0.843 |  |  |  |
| - | + | 176 | 64 | 1.618 |  |  |  |
| + | + | 843 | 272 | 1.436 |  |  |  |
| 1. **Interactions Between Trans Fatty Acids (TFAs) and Alpha-linolenic** **Acids (ALAs)** | | | | | | | |
| TFA | ALA | Non-atopic Non-AD Controls | Moderate-to-severe AD | Odds Ratio | SF P Value | SF  (95% CI) | Interaction |
| - | - | 1053 | 225 | Reference | 0.411 | 0.817  (0.504-1.323) | NA |
| + | - | 213 | 48 | 1.055 |  |  |  |
| - | + | 147 | 55 | 1.751 |  |  |  |
| + | + | 872 | 281 | 1.508 |  |  |  |
| 1. **Interactions Between Saturated Fatty Acids (SFAs) and Monounsaturated Fatty Acids (MUFAs)** | | | | | | | |
| SFA | MUFA | Non-atopic Non-AD Controls | Moderate-to-severe AD | Odds Ratio | SF P Value | SF  (95% CI) | Interaction |
| - | - | 1036 | 237 | Reference | 0.942 | 1.020  (0.608-1.711) | NA |
| + | - | 217 | 46 | 0.927 |  |  |  |
| - | + | 114 | 40 | 1.534 |  |  |  |
| + | + | 899 | 298 | 1.449 |  |  |  |
| 1. **Interactions Between Saturated Fatty Acids (SFAs) and Linoleic Acids (LAs)** | | | | | | | |
| SFA | LA | Non-atopic Non-AD Controls | Moderate-to-severe AD | Odds Ratio | SF P Value | SF  (95% CI) | Interaction |
| - | - | 1004 | 225 | Reference | 0.619 | 0.890  (0.561-1.410) | NA |
| + | - | 249 | 58 | 1.039 |  |  |  |
| - | + | 160 | 57 | 1.590 |  |  |  |
| + | + | 853 | 281 | 1.470 |  |  |  |
| 1. **Interactions Between Saturated Fatty Acids (SFAs) and Alpha-linolenic** **Acids (ALAs)** | | | | | | | |
| SFA | ALA | Non-atopic Non-AD Controls | Moderate-to-severe AD | Odds Ratio | SF P Value | SF  (95% CI) | Interaction |
| - | - | 1052 | 230 | Reference | 0.164 | 0.714  (0.445-1.148) | NA |
| + | - | 201 | 53 | 1.206 |  |  |  |
| - | + | 148 | 56 | 1.731 |  |  |  |
| + | + | 865 | 282 | 1.491 |  |  |  |
| 1. **Interactions Between Monounsaturated Fatty Acids (MUFA) and Linoleic Acids (LAs)** | | | | | | | |
| MUFA | LA | Non-atopic Non-AD Controls | Moderate-to-severe AD | Odds Ratio | SF P Value | SF  (95% CI) | Interaction |
| - | - | 1117 | 258 | Reference | 0.341 | 0.724  (0.373-1.407) | NA |
| + | - | 96 | 29 | 1.308 |  |  |  |
| - | + | 64 | 22 | 1.488 |  |  |  |
| + | + | 974 | 317 | 1.409 |  |  |  |
| 1. **Interactions Between Monounsaturated Fatty Acids (MUFA) and Alpha-linolenic** **Acids (ALAs)** | | | | | | | |
| MUFA | ALA | Non-atopic Non-AD Controls | Moderate-to-severe AD | Odds Ratio | SF P Value | SF  (95% CI) | Interaction |
| - | - | 974 | 229 | Reference | 0.509 | 0.861  (0.553-1.342) | NA |
| + | - | 239 | 58 | 1.032 |  |  |  |
| - | + | 195 | 70 | 1.527 |  |  |  |
| + | + | 843 | 269 | 1.357 |  |  |  |
| 1. **Interactions Between Linoleic Acids (LAs) and Alpha-linolenic** **Acids (ALAs)** | | | | | | | |
| LA | ALA | Non-atopic Non-AD Controls | Moderate-to-severe AD | Odds Ratio | SF P Value | SF  (95% CI) | Interaction |
| - | - | 978 | 233 | Reference | 0.875 | 1.039  (0.646-1.672) | NA |
| + | - | 228 | 51 | 0.939 |  |  |  |
| - | + | 163 | 53 | 1.365 |  |  |  |
| + | + | 905 | 287 | 1.331 |  |  |  |

(+) denotes a high estimated total fatty acids amount in diets, while (-) denotes a low estimated total fatty acids amount in diets.

CI denotes confidence interval.
